# Patient Controlled Intravenous Analgesia with Oxycodone or Sufentanil After Hip Surgery: Study Protocol for a Multi-Centered, Randomized Controlled Trial

**DOI:** 10.3390/jcm14051525

**Published:** 2025-02-25

**Authors:** Chenxi Liao, Haibei Zhu, Jie Zhong, Xingning Lai, Boyi Zhang, Ren Liao

**Affiliations:** 1 Department of Anesthesiology, West China Hospital, Sichuan University, Chengdu 610041, China; 2 Research Unit for Perioperative Stress Assessment and Clinical Decision, Chinese Academy of Medical Sciences (2018RU012), West China Hospital, Sichuan University, Chengdu 610041, China; 3 Division of Anesthesiology & Perioperative Medicine, Singapore General Hospital, Outram Road, Singapore 169608, Singapore; 4 Department of Anesthesiology, Hainan General Hospital, Road Xiuhua, Haikou 570100, China

**Keywords:** randomized controlled trial, oxycodone, sufentanil, patient-controlled intravenous analgesia, hip surgery

## Abstract

**Background**: Patient-controlled intravenous analgesia (PCIA) after hip surgery should be focused on sufficient analgesia, recovery, and the risk of adverse effects. Sufentanil PCIA offers effective analgesia but with obvious side effects. Oxycodone, a semi-synthetic opioid, is reported to have good analgesic effects with fewer adverse effects compared to strong opioids. We hypothesize that in hip surgery, compared with sufentanil PCIA, oxycodone PCIA in an equipotent dose to sufentanil could achieve similar postoperative analgesia while reducing the incidence of adverse effects associated with strong opioids. **Methods**: This multi-centered, randomized, controlled open-label clinical trial compares the efficacy of oxycodone and sufentanil for PCIA in hip surgery patients. **Results**: A total of 570 subjects will be randomly allocated in a 1:1 ratio into either the oxycodone group or sufentanil group. The primary outcome is the resting numerical rating scale (NRS) pain scores at 2 h after surgery. The secondary outcomes include the incidence of postoperative nausea and vomiting (PONV), NRS pain scores on movement, complications, mobilization time, length of hospital stay, total in-hospital cost, etc. **Conclusions**: This trial will provide evidence for the choice of PCIA in hip surgery by comparing the analgesic efficacy and side effects of oxycodone and sufentanil, serving as a foundation for postoperative pain management guidelines and recommendations. Trial Registration: Clinical Trials NCT03685188.

## 1. Introduction

Complex etiologies contributed to hip pathology such as chronic conditions including femoral head necrosis [1], primary hip dysplasia [2,3], hip joint osteoarthritis [4,5] and rheumatoid arthritis as well as acute trauma like femoral neck fractures and intertrochanteric fractures [6]. These are also common reasons for elderly patients and those with frailty requiring emergency surgery and hospitalization [7]. As the primary treatment for hip disease, hip surgery consists of closed reduction and internal fixation, open reduction and internal fixation, hemiarthroplasty (femoral head replacement) and total hip arthroplasty, etc. Over the past two decades, the incidence and prevalence of both chronic conditions such as hip osteoarthritis and acute conditions like hip fractures have significantly increased worldwide. The global incidence of hip osteoarthritis has risen from 740,000 cases annually to 1.58 million cases, representing a total increase of 115.40%. The age- and sex-standardized incidence rates of hip fractures have fluctuated between 95.2 and 315.9 cases per 100,000 population [8,9]. It is estimated that approximately 4.5 million people worldwide are disabled due to hip fractures each year, and this number is expected to increase to 21 million people over the next 40 years [6]. Extensive tissue damage, particularly bone and surrounding muscle connective tissue accompanied by hip surgery, could subsequently lead to severe pain, and a considerable number of patients suffered from analgesic under-administration and insufficient pain relief following the surgery [10].

Patient-controlled intravenous analgesia (PCIA), as an essential part of multimodal analgesia and an effective method for postoperative pain management, has already been widely applied in clinical practice and highly recommended for postoperative pain control. In terms of postoperative analgesia, opioid was not only regarded as the cornerstone but the gold standard for managing moderate-to-severe pain [11]. Sufentanil, a selective μ receptor agonist opioid, is characterized by its rapid onset, strong analgesic effects, and short duration and also has active metabolites, similar to morphine, algentanil, and fentanyl. It is the most commonly used opioid in intraoperative analgesia and PCIA after operation [12]. However, despite its widespread popularity among anesthesiologists, sufentanil carried notable side effects and risks, since μ receptors were also distributed in the respiratory centers in the brainstem, intestines, and chemotherapy trigger zones. The activation of μ receptors located in the above areas was closely related to postoperative nausea and vomiting (PONV), respiratory depression, and constipation. In addition, pruritus, cough, and sedation may also occur occasionally [13]. The PONV is particularly prevalent, affecting more than 30% of surgical patients, which can increase the risk of complications such as aspiration, esophageal rupture, pneumothorax, and wound dehiscence [14]. Furthermore, tolerance, dependence, addiction, and even hyperalgesia were also considered to be additional deleterious effects of opioid analgesics. Until now, efforts have been made to curb the abuse of opioid prescriptions and minimize the risks associated with it in the United States [15]. A previous study found that pain scores at different postoperative time points were comparable between the sufentanil and oxycodone groups and the time to the first occurrence of PONV was longer in the oxycodone group [14]. In a study involving elderly patients undergoing total hip arthroplasty with PCIA, the incidence of PONV and pruritus was significantly higher in the sufentanil group [16].

Acute postoperative pain is a challenge encountered by anesthesiologists, surgeons, and patients alike. The majority of patients undergoing surgery experience acute postoperative pain, with 80% reporting moderate-to-severe pain [17]. In addition, a higher proportion of older adults (>65 years) was observed among the patients undergoing hip surgery, and these patients tend to be accompanied by other late-stage comorbidities like hypertension, coronary disease, and diabetes, which put a greater burden on patients during the surgical procedure and recovery period [10,18,19]. On one hand, poorly managed pain leads to numerous adverse outcomes in elderly patients, including delayed recovery, increased risks of complications, and declined quality of life. On the other hand, patients with chronic comorbidities become more intolerable to the side effects of sufentanil, which leaves patients physically and psychologically devastated. All these negative adverse effects could impact the patient’s analgesic satisfaction and compromise the postoperative recovery process. It is essential to ensure adequate analgesia while minimizing related adverse effects perioperatively for such a group of patients, thereby promoting patients’ rehabilitation [20,21,22]. Oxycodone is a semi-synthetic opioid which is derived from thebaine. Unlike other strong opioids that selectively agonize the μ-opioid receptor, oxycodone also exerts additional agonistic effects on the κ and δ receptors, which may contribute to superior efficacy in acute pain management. Due to its relatively weaker affinity for the μ-opioid receptor, oxycodone may exhibit a lower incidence of opioid-related adverse effects compared to sufentanil in clinical practice [17]. In early studies by Silvas M. et al., intravenous oxycodone is considered to be of similar potency to intravenous morphine, with good analgesia and lower rates of adverse effects in patients undergoing superficial or orthopedic surgery [23]. Feng X. demonstrated in a meta-analysis that oxycodone PCIA is superior to sufentanil PCIA in alleviating pain during rest and movement. Additionally, significant differences were observed in terms of side effects, including pruritus, dizziness, postoperative nausea and vomiting (PONV), and respiratory depression [24]. Therefore, oxycodone may be a more reasonable and effective choice for PCIA. However, most current studies on oxycodone have concentrated on laparoscopic cholecystectomy and various abdominal surgeries, with the mean age of the participants studied ranging from 40 to 55 years old. Crucially, there are very few studies on oxycodone analgesia in major orthopedic surgery, and there is a lack of large sample studies at present [25,26,27].

Based on these existing studies, we hypothesize that in hip surgery, compared with sufentanil PCIA, the equipotency dose of oxycodone PCIA may provide comparable postoperative analgesic efficacy to sufentanil PCIA while also decreasing the incidence of adverse effects associated with strong opioids. To test this hypothesis, we designed this randomized controlled trial to compare the efficacy of oxycodone and sufentanil for PCIA in patients undergoing hip surgery and to identify an optimal postoperative analgesic regime with fewer adverse effects to promote early recovery after surgery (ERAS).

## 2. Materials and Methods

### 2.1. Study Objectives

This study aims to be jointly led by anesthesiologists and orthopedic surgeons, using a multi-center randomized controlled research method to compare the safety and efficacy of oxycodone and sufentanil in patient-controlled intravenous analgesia for hip surgery patients and to find a pain management plan that has fewer adverse effects and is beneficial for patients’ postoperative recovery.

### 2.2. Study Design

**Figure 1:**
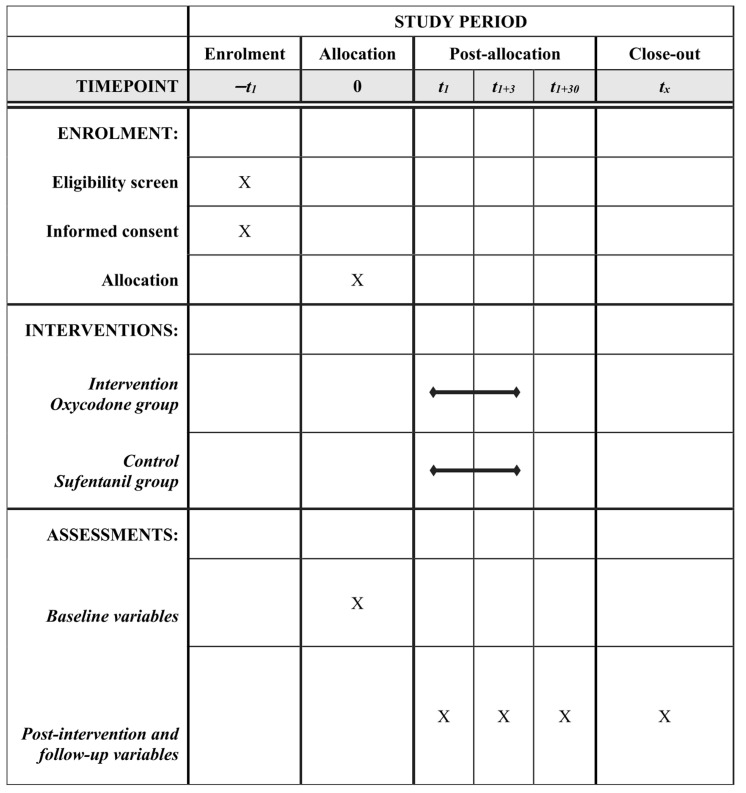
SPIRIT schedule of enrolments, interventions, and assessments.

**Figure 2:**
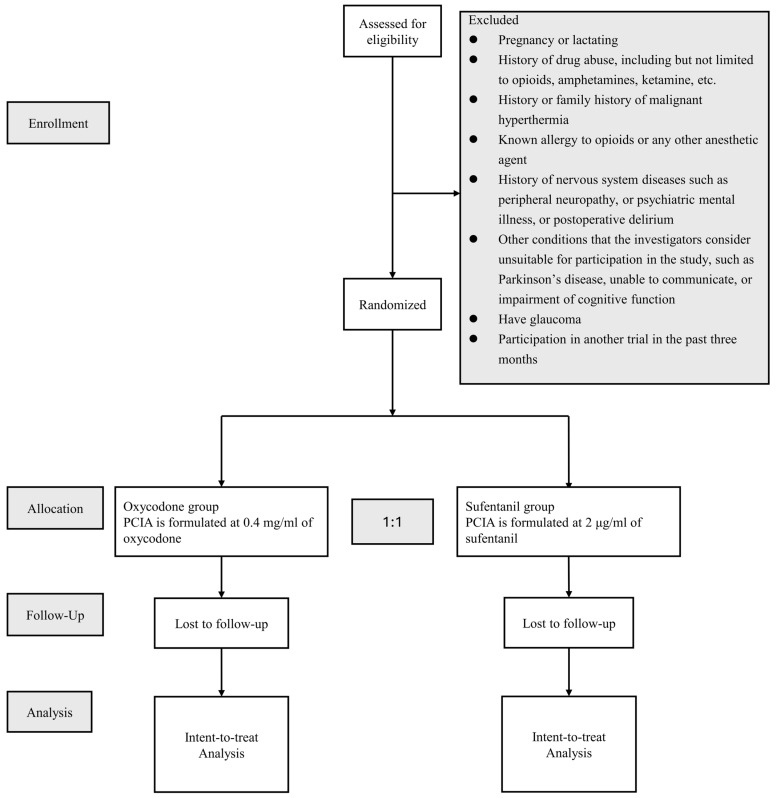
Flowchart for participant eligibility, interventions, follow-up, and analysis.

This trial is a multi-centered, open-label, randomized controlled clinical trial comparing the efficacy of oxycodone (Oxycodone Hydrochloride Injection, 10 mg/mL; Mundipharma, Beijing, China) and sufentanil (Sufentanil Citrate Injection, 50 μg/1 mL; Yichang Humanwell Healthcare, Wuhan, China) for postoperative PCIA in patients undergoing hip surgery, including total hip arthroplasty (THA), bipolar femoral head arthroplasty (BFHA), and proximal femoral nail antirotation (PFNA). This study will be executed in general hospitals in Chengdu, Sichuan, and Wuxi, Jiangsu, China. This study was approved by the Biological–Medical Ethical Committee of West China Hospital, Sichuan University (WCH201806). And this study was prospectively registered at www.clinicaltrials.gov (ClinicalTrials.gov Identifier: NCT03685188, accessed on 13 September 2018). It will be conducted according to the rules of the Declaration of Helsinki. The study protocol was conducted following the Standard Protocol Items: Recommendations for Interventional Trials (SPIRIT), which is provided in Figure 1 [28], and a brief flow diagram of the trial under the guidance of the Consolidated Standards of Reporting Trials (CONSORT) statement (http://www.consort-statement.org/, accessed on 15 September 2023) is summarized in Figure 2 [29].

### 2.3. Recruitment and Consent

Participants will be recruited from 1 March 2019, and a total of 570 patients undergoing hip surgery will be enrolled at West China Hospital of Sichuan University, Wuxi Traditional Chinese Medicine Hospital, First People’s Hospital of Longquanyi District, and Xindu District People’s Hospital of Chengdu. All patients scheduled for hip surgery will be screened on the day of hospitalization. We will assess the patients’ eligibility according to the pre-determined inclusion criteria and exclusion criteria. For the eligible patients, the researchers will explain the details of the study and inform the subjects that they are free to withdraw their consent at any time without any reason. Written informed consent forms will be obtained from each subject. The privacy of any participant will be strictly protected, and any confidential information will not be exposed throughout the process of the trial execution, data collection, and analysis stages.

### 2.4. Eligibility Criteria

Adult patients with American Society of Anesthesiologists (ASA) physical status I–III, scheduled for unilateral hip surgery, and who provided the “informed consent form” will be enrolled. We will further exclude patients if they are pregnant or lactating women, have a history of drug abuse (i.e., opioids, amphetamines, ketamine, etc.), are allergic to opioids or any other anesthetic agent, have a history or family history of malignant hyperthermia, have a history of nervous system diseases such as peripheral neuropathy, psychiatric mental illness, or postoperative delirium, have other conditions that the investigators consider unsuitable for participation in the study (e.g., Parkinson’s disease, inability to communicate, cognitive impairment, etc.), have glaucoma, or have participated in another trial in the past three months.

Exit criteria: (1) Participants enrolled in this study have the right to discontinue further involvement without any reason at any time, and the participation will be terminated immediately once the subject raises the request. (2) Conversely, the investigators have the authority to withdraw a participant at any point. If a subject develops any condition that aligns with the exclusion criteria during the study, or if the safety of the patient is jeopardized, the study will be halted immediately.

### 2.5. Randomization and Blinding

A Central Randomization System (CRS) will be applied to randomize the subjects (http://www.medresman.org.cn/login.aspx, accessed on 18 February 2025). After meeting the eligibility criteria and providing informed consent, each participant will be randomly assigned to either the sufentanil group or oxycodone group in a 1:1 ratio according to their enrollment order. Participants’ information will be entered into the CRS by the research coordinator, and the system will automatically generate a unique randomization number to complete the allocation, ensuring balance between the two groups at baseline and preventing bias. The randomization process will be recorded by the system for easy retrieval and verification, with access restricted to authorized personnel only. In addition, the process will be regularly monitored to ensure compliance with the study protocol and ethical standards.

This study was designed as an open-label RCT. After obtaining the random number of patients, the researchers will inform the research nurses to formulate the study drugs according to the study group allocation. In China, both sufentanil and oxycodone are strictly controlled opioid analgesics. Patients who receive treatment with these drugs must be registered with their real name, and these drugs must be marked and labeled on the patient-controlled analgesic pumps. The researchers, participants, and clinical staff will be aware of the group allocation, but the follow-up personnel and the statisticians will be blinded to the group allocation.

### 2.6. Sample Size Calculation

The sample size for this study was calculated using PASS 15.0 software. The primary hypothesis of this trial was that the analgesic effect of oxycodone PCIA is not inferior to the equipotency dose of sufentanil PCIA, and the incidence of PONV is lower in oxycodone PCIA than in sufentanil PCIA during the postoperative period. Therefore, the effect of postoperative analgesia is considered as the primary outcome and compared between groups with a non-inferiority design. The incidence of PONV is included as the most important secondary outcome and compared by superiority design. According to preliminary observational studies, the mean numerical rating scale (NRS) pain scores of patients receiving oxycodone PCIA and sufentanil PCIA at 2 h after operation were 3.5 and 3, respectively, with a common standard deviation of 0.866 [30,31]. We conservatively assumed that the difference in NRS between the two groups is 0 and the common standard deviation is 0.866. We set the noninferior margin as 1/10 of the mean value 0.3, and assuming the difference between two groups at a 2.5% significance level and a power of 0.80, 132 subjects are required in each group. Accounting for an estimated 20% dropout rate, 165 subjects per group, and a total of 330 subjects are required. For the secondary outcome, the incidence of PONV was reported to be 38.8% in sufentanil and 25.3% in oxycodone [14]. Assuming the difference between the two groups at a 2.5% significance level and a power of 0.80, 223 subjects are required in each group. Considering an estimated 20% dropout rate, 279 subjects in each group, and a total of 558 subjects are needed. Eventually, we plan to enroll 570 subjects in this study.

### 2.7. Perioperative Management

After admission, preoperative evaluations will be arranged in accordance with the routine procedures of participating hospitals. Vital signs including blood pressure, electrocardiogram, and pulse oximetry will be continuously monitored during operation. General anesthesia with sufentanil, atracurium, propofol, and remifentanil will be applied to the patients. During surgeries, the total dosage of sufentanil administrated by intravenous injection should be controlled between 0.3 and 0.5 μg/kg. And the maintenance of remifentanil (0.1~0.3 μg/kg/min) was regulated to keep hemodynamic alteration between 20% compared to the baseline blood pressure. Aside from sufentanil and remifentanil, no other concomitant analgesic drugs will be administered during the perioperative period, and remifentanil will be discontinued immediately upon skin closure before the end of the surgery. Indwelling catheters for postoperative analgesia should be avoided. Antiemetic drugs will not be given during operation, and 0.25% ropivacaine at 0.5 mL/kg for incision infiltration will be applied after operation.

### 2.8. Interventions

According to the analgesia formula of postoperative PCIA, the participants will be randomly divided into the oxycodone group or the sufentanil group, and both groups’ total volume of pump were 100 mL.

- Oxycodone group: PCIA is formulated at 0.4 mg/mL of oxycodone.
- Sufentanil group: PCIA is formulated at 2 μg/mL of sufentanil.

No background infusion will be applied for PCIA in both groups. When the participants experience pain, they can activate the analgesic pump to infuse 2 mL of analgesic drugs. The lock-out time is 12 min, and the pump can be activated up to 5 times per hour.

### 2.9. Acute Pain Rescue Measures

If pain persists or the NRS pain score exceeds 4 after 30 min of surgery, an initial bolus will be administered via the PCIA pump as a preliminary pain relief measure. If the pain score remains above 4 after 12 min, a second bolus will be given. If the pain score continues to exceed 4 thereafter, 5 mg of morphine will be considered as rescue analgesia. Before the implementation of rescue analgesia, the follow-up personnel will inquire about the times of bolus and the current pain score.

### 2.10. Safety Assurance

Throughout the study, we will rigorously document the occurrence of all complications and adverse events. In the event of any complication or serious adverse event (Table 1), prompt management will be initiated in accordance with clinical guidelines and emergency protocols, with the involvement of relevant specialist physicians. Additionally, the ethics committee will be notified in a timely manner. During hospitalization, the continuous monitoring of baseline vital signs will be conducted, and patients will be immediately withdrawn from the study if respiratory depression requiring intervention, fatal events, or complications exceeding Grade III occur.

### 2.11. Outcome Measures

The primary outcome and secondary outcomes are listed in Table 2.

### 2.12. Study Management

The implementation of the study and the data completeness and accuracy will be supervised by the Department of Anesthesiology of West China Hospital and the Office of Scientific Research at West China Hospital. Dr. Ren Liao, as the alert personnel, will be responsible for managing serious complications and making the final decision to terminate the trial. Baseline data collection and postoperative follow-up will be performed by clinical research coordinators. The data safety and monitoring board including an anesthesiologist, an orthopedic surgeon, an ethicist, a statistician, and an internal physician relevant to this trial, will be involved for the entire duration of the trial to review all investigational data for accuracy and completeness periodically to ensure protocol compliance. No preliminary analysis will be conducted before the completion of this study. All personnel will adhere to the principle of confidentiality and there are no conflicts of interest. Before the trial began, all members of the study team will receive specialized training relevant to the research.

### 2.13. Statistical Analysis

All outcomes will be analyzed by using SPSS20 software (Statistic Package for Social Science, SPSS, Inc., Chicago, IL, USA) with intent-to-treat analysis. The repeated measurement variance analysis will be used to compare the resting NRS pain scores at 30 min, 2 h, 6 h, 24 h, 48 h, and 72 h after the surgery, as well as the NRS pain scores on movement from day 1 to day 3 after operation. To address the issue of multiple comparisons, the Bonferroni correction methods will be used depending on the number of comparisons. The quantitative data will be tested for normal distribution, for example, the total hospitalization days, days of postoperative hospital stay, total hospitalization expenses, etc. The data in accordance with the normal distribution will be presented as mean ± standard deviation, and the analysis will be performed by using Student’s *t*-test. The data of the non-normal distribution will be presented as the median (minimum, maximal), and the comparison between groups will be performed by Kruskal–Wallis tests. The Z test will be used to compare the incidence of PONV, postoperative adverse reactions, and the incidence of re-admission within 30 days after surgery. The *t*-test will be used to compare the dosage of postoperative analgesics which have been used by the patients. *p* < 0.05 indicates a statistical difference.

For the primary outcome and the most important secondary outcome, we will perform subgroup analysis based on group differences. Subgroups are defined by age (<65, ≥65), gender (Male, Female), and acute trauma (from injury time to surgery time < 14 days) or chronic joint disease (disease course > 1 year). Analyses will be performed for each subgroup in a similar way to the primary analysis. Forest plots will be drawn based on the odds ratios and corresponding 95% confidence intervals.

### 2.14. Patient Involvement and Ethical Considerations

Patients and the public were not involved in the design, implementation, reporting, or dissemination plans of this study. However, they will meet with the investigators, have informative interviews and sign a consent form indicating their understanding of patient’s enrollment in the trial. Additionally, to enhance the control of opioid use and mitigate the risk of misuse, all patients will receive detailed pain management education prior to surgery. The educational content will cover the proper use of the analgesic pump, potential adverse effects and their management, as well as the gradual reduction in opioid use. Although this document is a research protocol, the dissemination and impact of the research will involve patient and peer support organizations and public policymakers. We would like to thank all participants.

In this study, postoperative pain management will be administered via PCIA pump, which delivers a controlled dose of oxycodone or sufentanil. NRS pain scores will be regularly assessed at multiple time points following surgery. The PCIA pump was configured with a fixed concentration of analgesic for ease of dosage calculation, allowing patients to self-administer analgesia by pressing the pump according to their individual pain requirements. However, to mitigate the risk of opioid dependence or misuse, it is recommended in clinical practice to gradually reduce opioid use in accordance with the patient’s postoperative recovery by extending the lock-out interval or reducing the activation times per hour.

### 2.15. Study Status and Timeline

The Biological–Medical Ethical Committee of West China Hospital, Sichuan University has approved this investigation. The version of the study protocol is 1.0 on 11 March 2018. The first investigators’ meeting was held on 1 December 2018. The recruitment of subjects started on 1 March 2019, and the first subject was enrolled on 7 March 2019. Recruitment is expected to be completed on 31 December 2024, and the expected completion date of the study is 31 July 2025.

## 3. Discussion

Pain is one of the major factors bringing uncomfortable feelings and impeding early recovery after hip surgery. The presence of poorly controlled pain may cause postoperative adverse outcomes such as the prolonged duration of immobilization, poor wound healing or infection, a higher risk of thromboembolism, and delayed postoperative rehabilitation, especially for elderly patients [14,32]. PCIA, with efficient analgesic effects, plays an important role in the multimodal analgesia regime and has been widely recommended by several guidelines and expert consensus. However, multiple complications of PCIA associated with strong opioids like fentanyl and sufentanil have limited the clinical application and even become drawbacks of PCIA [33]; for example, less than 5% of patients who received orthopedic surgery accepted PCIA with over 10,000 surgical cases performed annually in our department of orthopedics. Most patients would rather bear the pain than experience nausea, vomiting, or dizziness.

Oxycodone is not only a μ-opioid receptor agonist but also activates κ and δ receptors in the central nervous system, peripheral nervous system, and autonomic nervous system. Compared to pure μ-opioid receptor agonists, oxycodone may offer better postoperative pain relief with less opioid-related complications due to its unique mechanism [14,30,31,34]. However, previous studies have reported inconclusive results comparing the application of oxycodone and sufentanil in PCIA, the limitations of which remained obvious. Han et al. and Dang et al. [30,31] found that oxycodone PCIA provided the same postoperative pain relief compared to sufentanil PCIA but was accompanied by the lower consumption of analgesic drugs and fewer adverse effects. In the two studies, the continuous infusion mode with PCIA contributed to accumulated opioid consumption leading to increased risks of PONV, constipation, respiratory depression, and so on. Another randomized controlled trial comparing oxycodone and sufentanil PCIA with 50 participants scheduled for laparoscopic radical gastrectomy found that, although no significant differences in VAS scores for resting and coughing or in the incidence of adverse effects within 48 h post-operation, patient satisfaction was notably higher in the oxycodone group [35]. Utilizing antiemetics before the end of the surgery and limited sample size seem like the reasons for discrepancy. Other studies on oxycodone predominantly focused on visceral pain controlment and the comparison of other opioids, and scarce studies have been conducted to compare oxycodone and sufentanil for PCIA in hip surgery, let alone large-sample studies [25,36,37]. Therefore, we designed the current study to address the aforementioned gaps and aim to supply more comprehensive and reliable evidence of oxycodone PCIA utilization in hip surgery. Given the ongoing opioid epidemic, it is critical to explore alternative analgesic options that can deliver effective pain relief while minimizing opioid-related adverse effects. By publishing this protocol, we aim to provide evidence that could influence clinical practices, offering a foundation for guidelines and recommendations for the management of postoperative pain in hip surgery. Furthermore, the findings of this study have the potential to catalyze future research into opioid-sparing strategies, particularly for high-risk patient populations. This research may contribute to shaping the broader landscape of opioid management, promoting safer analgesic options and aiding in the reduction in opioid dependence and misuse in postoperative care. Additionally, it will address the urgent need for evidence-based approaches that balance effective pain control with patient safety.

Achieving successful enhanced recovery after surgery (ERAS) relies on effective pain management and early mobilization [38,39,40]. On one hand, as previously mentioned, the frequency of medication administration due to postoperative acute pain could be lessened by oxycodone with more enduring half-life, which significantly improves postoperative pain satisfaction and comfort while reducing the risk of opioid-related adverse effects such as nausea and vomiting associated with opioids. Consequently, a virtuous circle of the early mobilization and recovery process seems to be promoted by oxycodone PCIA. On the other hand, providing adequate pain relief and minimizing side effects alone are insufficient; preserving the patient’s ability to move also occupies an essential position for ERAS. Although epidural or nerve block techniques, such as fascia iliaca block or femoral nerve block, can provide reliable analgesic effects, the associated motor block, which induces weakened lower limb muscle strength and limits mobilization, cannot be ignored [41]. Therefore, in this trial, intravenous PCA is chosen to conserve muscle strength.

In light of the latest published pain management guideline, adopting background infusion in PCIA is not recommended, as various evidence suggests no improved analgesic effect compared to PCIA without background infusion. In addition, background opioid infusion was considered to potentially associate with PONV or respiratory depression [42]. So, we changed the mode of PCIA from continuous infusion (background analgesia) to PCIA without background analgesia. Theoretically, this model should decrease the occurrence of adverse effects. In this study, we included patients undergoing three types of hip surgeries, each varying in complexity and surgical duration. Given the potential impact of surgical duration on outcomes, we did not include it as a variable in our analysis. This decision was based on the randomized and balanced allocation of patients, which minimizes the likelihood that differences in surgical duration would influence the study results. As such, we do not anticipate significant confounding effects from surgery duration. Future studies may consider exploring the potential impact of surgical duration in more detail, particularly if a broader range of surgical procedures with varying durations are included.

This study has several inevitable limitations. Firstly, given that both sufentanil and oxycodone are strictly controlled analgesics in China, both participants and researchers will be aware of the drugs being used. Thus, the open-label design may lead to ascertainment bias, allocation bias, or measurement bias, potentially affecting patient inclusion, exclusion, and outcome analysis stages. However, we have implemented several measures to minimize these potential biases. Specifically, the follow-up personnel and statisticians will be blinded to the group allocations, multi-center data collection will be employed to enhance the external validity, and all researchers will undergo standardized training to ensure the consistent understanding and application of the assessment scales. Second, our study only tested and compared specific doses of sufentanil and oxycodone; in other words, the results may not fully represent all possible analgesic doses. Further studies are demanded to determine whether different doses affect the trial outcomes. Third, existing data have indicated that significant individual variability could be observed in the requirement for adequate pain relief with oxycodone. Therefore, more detailed subgroup analyses are necessary to control for and analyze confounding factors. Fourth, the dosages of sufentanil and oxycodone were not specifically adjusted based on the patient’s weight (mg/kg) in our study. Instead, each patient’s analgesia pump was set to a fixed drug concentration, allowing patients to self-administer bolus doses in accordance with their individual pain assessments and requirements. This method may have contributed to variability in the effective dosage across different patients. Future studies could investigate the potential advantages of weight-based dosing to better assess its impact on analgesic efficacy and safety. Finally, our study planned to include a total of 550 patients from four centers in Chengdu, Sichuan, and Wuxi, Jiangsu, which only represents a subset of broader populations in China. To enhance the reliability and generalizability of the trial results, it would be beneficial to expand the sample size and include a more diverse population.

## Figures and Tables

**Table 1:** Definition of main postoperative adverse events and management.

Postoperative Adverse Events	Definition or Clinical Symptoms	Management
Nausea/vomiting	Symptoms of nausea and/or vomiting	Metoclopramide (iv) at 10 mg or Tropisetron (iv) at 5 mg or Ondansetron (iv) at 4 mg.
Pruritus	Definition: An itching sensation that induces rubbing or scratching the skin for relief. Mild: Patients can tolerate it, so it does not require medication. Moderate to severe: Cannot tolerate and require medication.	Mild: Skin moisturization or application of Vaseline. Moderate to severe: Nalbuphine (iv) at 5 mg or Ondansetron (iv) at 4 mg or Loratadine (os) at 10 mg.
Dizzy	A range of sensations, such as feeling faint, woozy, weak, or unsteady.	Bed rest and find the cause of your condition and your symptoms.
Respiratory depression	SpO2 < 90% or breathing rate less than 8–10 breaths/min.	Stop use of PCIA pump, give oxygen, keep the airway open, and give nalmefene (0.25 mcg/kg) if necessary.

**Table 2:** The details of outcome measures.

Measures	Description
Primary outcome	The postoperative resting numerical rating scale (NRS) pain scores at 2 h after surgery:
Secondary outcomes	The incidence of PONV, which is defined as the proportion of subjects who experienced PONV.
	The severity of the first PONV and the most severe PONV, which is scored from 0 to 10, and 0 represents no PONV at all, and 10 represents very severe PONV.
	Time from the end of operation to the first onset of PONV (hours).
	Postoperative resting NRS pain scores at 30 min, 6 h, 24 h, 48 h, and 72 h after the surgery.
	Postoperative NRS pain score on movement 3 days after operation.
	Postoperative complications, which are divided into five grades: (1) Grade I: Recovery after temporary treatment, e.g., postoperative anxiety, insomnia. (2) Grade II: Prolonged hospitalization, e.g., pulmonary infection requiring antibiotics or other treatment, surgical wound infection requiring wound debridement. (3) Grade III: Life-threatening complications requiring intense treatment during hospitalization and resulting in good functional recovery, e.g., dialysis therapy for acute renal insufficiency, mechanical ventilatory support for respiratory failure, or postoperative bleeding requiring re-operation. (4) Grade IV: Life-threatening complications resulting in a significantly decreased quality of life, e.g., myocardial infarction, stroke that left patient with paralytic limbs. (5) Grade V: All-cause mortality by 30 days after operation.
	Range of motion of hip joints 3 days after operation.
	Straight leg raising time, defined as the time from the end of operation to the time that the patient can raise his affected lower limb by himself (unit: hour).
	Ground exercise time, defined as the time from the end of operation to the time that a patient can do the ground exercise by himself (unit: hour).
	Mobilization time, which is defined as the time frame from the end of operation to being able to walk without external assistance (walking aids such as crutches can be used, unit: hour).
	Residual amount of drug in the analgesic pump.
	Postoperative analgesics requirement 3 days after operation.
	Total in-hospital cost.
	Length of stay (LOS) in hospital, which is defined as the time frame from the day of hospital admission to discharge from the hospital (unit: day).
	Postoperative hospital stay, which is defined as the time frame from the day of operation to discharge from the hospital (unit: day).
	Readmission rate by 30 days after discharge from the hospital.

NRS, numerical rating scales; PONV, postoperative nausea and vomiting; LOS, length of stay.

## Data Availability

At present, the datasets are not readily available. The individual participant data with anonymity will be uploaded on the IPD sharing platform to achieve the data sharing and will be available after the principal investigator agrees on reasonable request.

## List of Abbreviations

ASA	American Society of Anesthesiologists
CONSORT	Consolidated Standards of Reporting Trials
CRS	Central Randomization System
ERAS	Enhanced recovery after surgery
THA	Total hip arthroplasty
BFHA	Bipolar femoral head arthroplasty
PFNA	Proximal femoral nail antirotation
LOS	Length of stay
NRS	Numerical rating scales
PACU	Post-anesthesia care unit
PCA	Patient-controlled analgesia
PCIA	Patient-controlled intravenous analgesia
PONV	Postoperative nausea and vomiting
SPIRIT	Standard Protocol Items: Recommendations for Interventional Trials
SPSS	Statistic Package for Social Science Acknowledgements Special thanks to all participants in this study.
